# Genesis, controls and risk prediction of H_2_S in coal mine gas

**DOI:** 10.1038/s41598-021-85263-w

**Published:** 2021-03-11

**Authors:** Weidong Xie, Hua Wang, Meng Wang, Ye He

**Affiliations:** 1 Key Laboratory of Tectonics and Petroleum Resources of Ministry of Education, China University of Geoscience, Hubei, 430074 Wuhan China; 2 School of Earth Resources, China University of Geoscience, Hubei, 430074 Wuhan China; 3 School of Resources and Earth Geosciences, China University of Mining and Technology, Jiangsu, Xuzhou, 221116 China

**Keywords:** Chemical biology, Natural hazards

## Abstract

Abnormal H_2_S concentration in coal mine gas is a serious threat to normal mining activities, which has caused serious loss of life and property in many coal mines. This study explores the genesis and influencing factors of abnormal H_2_S concentration in coal mine gas, taking the Xishan coal mine in the Fukang mining area as a case study. The H_2_S formation by bacterial sulfate reduction (BSR) is simulated with a bacterial culture experiment and that by thermochemical sulfate reduction (TSR) is simulated with a thermal reduction experiment. The potential for a magmatic genesis is assessed using data regarding the tectonic evolution and history of magma intrusion in the study area. The factors influencing H_2_S formation and enrichment are then analyzed by a comprehensive consideration of the characteristics of coal, the gas composition, the coal seam groundwater geochemistry and other geological factors in the study area. The results show that the study area meets the necessary conditions for the BSR process to operate and that there is widespread BSR derived H_2_S. TSR genesis H_2_S mainly forms in coal fire areas and their vicinity, while there is little contribution from magmatically formed H_2_S. The concentration of H_2_S is negatively correlated with the buried depth of the coal seam, the concentrations of CH_4_, N_2_ and CO_2_, and the ash yield; and it is positively correlated with the volatiles yield and total sulfur content. In addition, in areas with abnormally high H_2_S concentration, the concentration of SO_4_^2−^ is obviously lower, HCO_3_^−^ + CO_3_^2−^ concentration is higher, and the HCO_3_^−^/SO_4_^2−^ value is larger than that in non-anomalous areas. Geologically, H_2_S enrichment is found to be controlled by lithology, tectonism, and hydrogeological conditions. Moreover, the results of predictive modeling show that areas prone to abnormal H_2_S concentration are generally spatially correlated with coal fire areas. In this study, the genetic types of H_2_S and the factors controlling their formation and retention are discussed, producing research results that have guiding significance for the prediction and prevention of the coal mine disasters that arises from abnormal H_2_S concentration.

## Introduction

H_2_S is a colorless, highly toxic gas with an unpleasant odor. The Chinese "Coal Mine Safety Regulations" states that the H_2_S concentration of coal mine gas is considered abnormal if it concentration is greater than 6.6 ppm^1–3^. Such concentrations present a serious threat to life and property in well mines due to the risk of H_2_S outburst^3–6^. In recent years, more than 10 people have died in H_2_S disasters in mines in Xinjiang, Sichuan, Hunan, Chongqing and elsewhere in China, and H_2_S poisoning incidents have led to shutdowns in Shandong, Shanxi and Henan provinces^4–7,22^. The strong odor of rotten eggs appeared in many of the main coal seams during the exploration and mining of the Xishan coal mine in the Fukang mining area, Urumqi, indicating that there is a potential risk of abnormal concentration of H_2_S, and the H_2_S disasters have ever resulted in both poisonings and fatalities. The resulted in the loss tens of thousands of day production during suspensions and seriously affected the safety of production^8–10^. It is essential to determine the genesis and controlling factors of abnormal H_2_S concentration as theoretical principles on which to base H_2_S-disaster prevention and control measures in mining areas. It is generally believed that the genesis of H_2_S in coal mines can be divided into four main types: bacteria sulfate reduction (BSR), thermochemical sulfate reduction (TSR), thermochemical thermal destruction of sulfur compounds (TDS) and magmatism,which of this genetic types dominates depends on geological setting^6,9,11–16^. Previous researches have indicated that the enrichment rule of H_2_S is similar to that of coal bed methane, being controlled by geological structure, the roof and floor lithology, hydrogeological conditions, and other factors^7,11,17–23^. A certain progress has been made in research into H_2_S disasters in coal mines in recent years, nevertheless, several key problems still require urgent consideration. For example, more research work is needed on the simulation, detailed characterization and identification of the factors influencing the formation process of H_2_S in coal mines. This study aimed to address these deficiencies in the literature by taking the Xishan mine as its research area, taking the physical characteristics of coal, geochemical conditions and other geological factors as its variables, and taking experimental simulation and theoretical analysis as its means, to reveal the genesis and controlling factors of H_2_S abnormal concentration in the study area. Moreover, it establishes a corresponding model, to predict the region in which abnormally H_2_S concentrations will develop, aiming to address the threat to safe production from H_2_S anomalies in both theoretical and practical aspects.

## Samples, experiments and theory

### Sample collection

**Figure 1:**
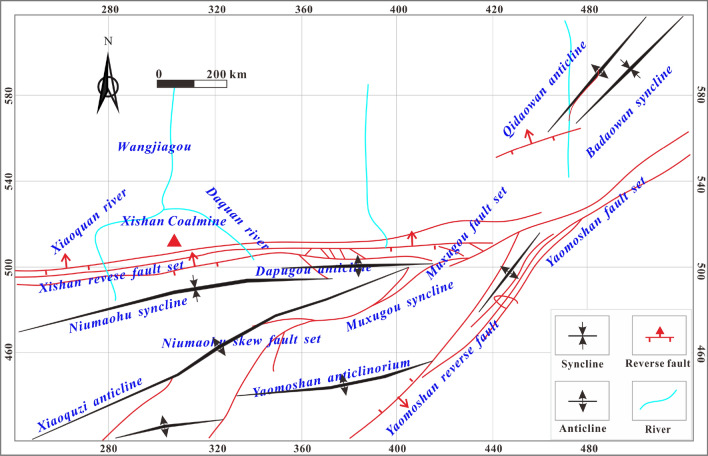
The structural position of the Xishan mine.

Xishan mine belongs to the Fukang mining area, Urumqi, western China Fig. 1. Sixteen matched coal and sixteen gas samples were collected from different buried depths for this study. The coal samples were characterized by block integrity and macro coal components, which can reduce the error caused by the components discrepancies of coal samples. The samples were sealed and transported to the laboratory immediately. Gas samples were collected by air bag (Polyvinyl chloride bag) method. The vacuum sealing bags were carried to mining face underground, and the gas samples were collected, sealed and immediately transported to the laboratory. In addition, ten groundwater samples were collected from coal seams (XJ-S10 and XJ-S9 from B_7_, XJ-S8 and XJ-S7 from B_8_ coal seam, XJ-S6 and XJ-S5 from B_11_ coal seam, XJ-S4 from B_12_ coal seam, XJ-S3 from B_14_ coal seam, XJ-S2 from B_15_ coal seam, and XJ-S1 from B_18_ coal seam). Among them, four of these were from normal areas and six from areas with abnormal H_2_S concentration.

### Experimental scheme

#### Basic data acquisition

Sixteen gas samples were tested by gas chromatography, and their gas composition was analyzed with an HP5890A high-sensitivity gas chromatograph produced by Hewlett-Packard, USA. The quality of sixteen coal samples was analyzed, and the industrial composition of each coal seam was tested. These experiments were carried out at the Jiangsu Institute of Geology and Mineral Resources using standards GB/T 30431-2013 and GB212-91, respectively. The ion composition, acidity, and alkalinity of the 10 coal seam groundwater samples were tested at Xinjiang Coal Design and Research Institute to characterize their geochemical characteristics, using ICS900 ion chromatograph manufactured by Diane company, America.

#### Simulation of BSR-genesis H_2_S

Sulfate-reducing bacteria (SRB) enrichment and culture experiments were carried out on groundwater samples (Sample XS-10 from the abnormal area of H_2_S concentration and sample XS-1 from the normal area of H_2_S concentration) from coal seams in area with normal and abnormal H_2_S concentrations. The culture medium comprised 4.5 g Na_2_SO_4_, 1 g NH_4_Cl, 0.5 g K_2_HPO_4_, 0.2 g MgSO_4_·7H_2_O, 0.06 g CaCl_2_, 0.1 g yeast extract, 1000 mL distilled water, and 3 mL sodium lactate. Its pH ranged from 6.5 to 7.2, a weakly alkaline environment. The culture medium was placed in a sterilizing pot for 20 min at a pressure of 0.2 MPa, and a temperature 121 °C; after which the sterilizing pot and the bacterial culture medium were cooled to room temperature. FeSO_4_·7H_2_O 0.5 g was then added to the culture medium, and the pot was then filled with N_2_ for 30 min.

A mixture of 2/3 volume culture medium and 1/3 coal seam groundwater from a normal and abnormal area were placed in two 1000 ml culture bottles, respectively, and cultured in a water bath at a constant temperature of 37 °C. When the water sample bottles were opened during the experiment, the strong odor of strong rotten eggs characteristic of H_2_S was emitted by bottles incorporating groundwater from H_2_S abnormal areas, and the liquid in the bottle rapidly turned black. After having been left to stand for one day, a solid black precipitate was found at the bottom of the bottle, while no obvious odor or experimental phenomena were observed in the culture bottle samples for the normal area. The redox potential and pH value of the culture solution in the bottle were measured every day. After the third day, there was an obvious slowdown in the reaction process.

Subsequent tests were carried out on the bacterial communities in the two types of culture bottles, including macro genome and 16 rDNA sequencing (V3-V4 amplification region; Miseq Miseq × 300 bp sequencing platform) and analysis by PHI5000 Versaprobe III X-ray photoelectron spectroscopy (XPS, manufactured by ULVAC company, Japan) of the solid sediments metabolized by bacteria. The relevant experiments were carried out at Shanghai Biotechnology Services Co., Ltd.

#### Simulation of TSR-genesis H_2_S

In order to explore the formation conditions and process of TSR-genesis H_2_S, experiments were conducted to simulate the thermal reduction of H_2_S. Coal samples (Which collected from the main mining coal seam, B_8_ coal seam with a buried depth of 242.79 m) and MgSO_4_, NaCl and deionized water were added to a fully enclosed autoclave reaction system. Eight temperature points were set in the experiment: 250 °C, 300 °C, 350 °C, 400 °C, 450 °C, 500 °C, 550 °C and 600 °C. The temperature was raised to the designated temperature point at a rate of 20 °C/h and was kept at each temperature point for 48 h. After the reaction had occurred, the gas was collected, and the H_2_S concentration was determined with a chromatograph.

## Results

### Basic experimental data

The results of gas chromatography and coal quality analysis are shown in Table 1. The sampling depth ranged from 237 to 364 m, and the main components of the gas samples were CH_4_, CO_2_, N_2_, and H_2_S. The concentration of CH_4_ ranged from 11.08% to 60.95%, with an average of 30.48%, the concentration of CO_2_ ranged from 4.4% to 13.77%, with an average of 7.77%, the concentration of N_2_ ranged from 15.91% to 75.92%, with an average of 46.32%, and the concentration of H_2_S ranged from 0.6% to 15.51%, with an average of 4.74%. The moisture content ranged from 1.05% to 2.05%, with an average of 1.48%, the volatile yield ranges from 23.97% to 41.12%, with an average of 34.34%, the ash yield ranged from 14.05% to 28.99%, with an average of 16.44%, and the total sulfur content ranged from 0.21% to 1.75%, with an average of 0.71%.

**Table 1:** Gas composition and the results of coal quality analysis.

Coal seam ID	Buried depth (m)	Gas content (cm^3^/g, ad)				Gas composition (mol%)				Coal quality (wt.%)			
		CH_4_	CO_2_	N_2_	H_2_S	CH_4_	CO_2_	N_2_	H_2_S	M_ad_	V_daf_	A_d_	S_t.d_
B_7_	411.61	4.51	0.85	4.65	0.15	44.36	8.41	45.73	1.5	1.05	23.97	14.05	0.41
B_7_	416.65	5.41	1	2.35	0.12	60.95	11.29	26.44	1.32	1.49	24.07	15.47	0.29
B_7_	419.32	3.54	0.67	5.33	0.13	36.84	6.92	55.4	1.4	1.32	34.85	16.31	0.35
B_8_	242.79	2.18	0.61	1.97	0.16	43.25	12.21	39.2	3.1	2.05	30.7	16.77	0.21
B_8_	250.18	3.16	0.76	5.11	0.53	31.06	7.46	50.22	5.24	1.36	34.26	21.01	0.71
B_8_	255.66	2.67	0.71	3.19	0.3	37.3	9.89	44.58	4.14	1.71	32.44	18.84	0.46
B_12_	167.3	1.52	0.26	2.68	0.93	25.36	4.4	44.51	15.51	1.31	38.5	16.51	0.95
B_12_	515.9	2.75	0.71	4.67	0.22	31.91	8.23	54.28	2.53	1.54	35.42	20.5	0.59
B_12_	516.2	2.15	0.48	3.71	0.68	28.64	6.32	49.4	9.02	1.43	36.96	18.51	0.77
B_14_	123.08	1.62	0.38	3.97	0.32	24.73	5.75	60.73	4.9	1.5	33.1	17.03	0.82
B_14_	361.03	1.34	0.68	6.22	0.4	16.33	8.32	75.92	4.9	1.15	35.73	12.65	0.72
B_15_	328.68	3.65	0.39	1.13	0.67	51.32	5.52	15.91	9.37	1.61	38.97	5.78	0.69
B_15_	103.13	0.47	0.28	2.99	0.03	11.08	6.6	70.89	0.6	1.77	41.12	28.99	1.27
B_15_	155.8	1.79	0.36	2.66	0.28	30.14	6.09	44.85	4.75	1.69	40.1	18	0.75
B_11_	172.01	2.75	0.26	2.63	0.36	33.79	3.14	32.38	4.43	1.28	34.94	6.22	0.69
B_18_	347.72	5.94	2.14	4.75	0.48	38.22	13.77	30.59	3.1				1.75

*ad* air-dried basis, *M*_*ad*_ moisture of air-dried basis, *V*_*daf*_ volatile of dry ash-free basis, *A*_*d*_ ash of dry basis, *S*_*t.d*_ total sulfur of dry basis.

The geochemical characteristics of the coal seam groundwater samples are shown in Table 2. The pH value of the water samples ranged from 6.95 to 8.3, with an average of 7.59, SO_4_^2−^ content ranged from 237.08 mg/L to 6676.2 mg/L, with an average of 2179.77 mg/L, HCO_3_^−^ + CO_3_^2−^ content ranged from 137.42 mg/L to 1888 mg/L, with an average of 682.82 mg/L. The concentration of SO_4_^2−^ ion ranged from 17.12% to 88.30%, with an average of 55.69%, HCO_3_^−^ + CO_3_^2−^ ion concentration ranged from 1.61% to 54.75%, with an average of 22.38%, and the value of γHCO_3_^−^/γSO_4_^2−^ ion ranges from 4.92% to 182.48%, with an average of 60.72%.

**Table 2:** Hydrogeological characteristics of coal seam water.

Sample ID	Percentage of ion concentration				γHCO_3_^−^/γSO_4_^2−^	pH	H_2_S concentration
	SO_4_^2−^		HCO_3_^−^ + CO_3_^2−^				
	mg/L	%	mg/L	%	%		
XJ-S1	6676.2	85.12	503.4	6.42	5.94	8.1	Normal
XJ-S2	2497.6	75.85	562.6	17.09	17.73	8.3	
XJ-S3	4841.4	73.18	1003.2	12.47	16.31	7.95	
XJ-S4	2198.36	88.30	137.42	1.61	4.92	7.8	
XJ-S5	1717.2	66.77	399.74	31.35	36.93	7.29	Abnormal
XJ-S6	598.47	57.64	806.26	31.35	52.54	7.29	
XJ-S7	237.08	29.78	435.87	54.75	135.6	7.37	
XJ-S8	998.71	39.55	525.71	20.82	58.01	7.6	
XJ-S9	1537	17.12	1888	21.03	96.69	6.95	
XJ-S10	495.67	23.58	565.95	26.92	182.48	7.22	

### Experimental simulation of BSR-genesis H_2_S

The experiment simulation of BSR indicated that the culture bottle containing groundwater from abnormal area promoted the formation H_2_S; and that SRB activity was strongest when the temperature was less than 50 °C and the pH conditions were neutral or weakly alkaline.

**Figure 2:**
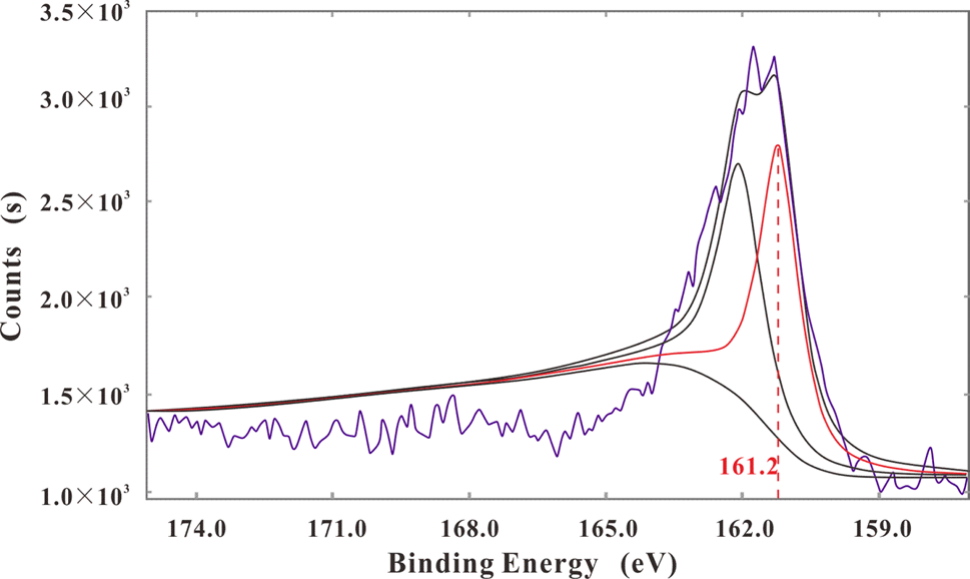
The peak spectrum of S of the black solid in the culture bottle with sample XS-10.

The results of macro genome and 16 rDNA sequencing showed that sulfate reducing bacteria (SRB) in the abnormal-area bottle included a large number of *Desulfovibrio* and *Desulfomicrobium* in two genera but that there were few methane-reducing bacteria. Thus it is inferred that the formation of BSR-genesis H_2_S was mainly promoted by *Desulfovibrio* and *Desulfomicrobium*. While a large number of methane-reducing bacteria were detected in the normal-area bottle, there were very few SRB. The similarity between the experimental results and the NCBI (National Center for Biotechnology Information) database was 95%-99%. The composition state of S in the black solid in the abnormal-area culture bottle was investigated by XPS narrow scanning surface analysis Fig. 2. The detection data were processed by XPS peak-separation software, and the binding energy was found to peak at 161.2 eV, indicating that the black precipitate was FeS. Namely, S^6+^ in SO_4_^2−^ was reduced to S^2−^ by SRB and combined with Fe^2+^ to form the solid black sediment.

### Experimental simulation of TSR-genesis H_2_S

**Figure 3:**
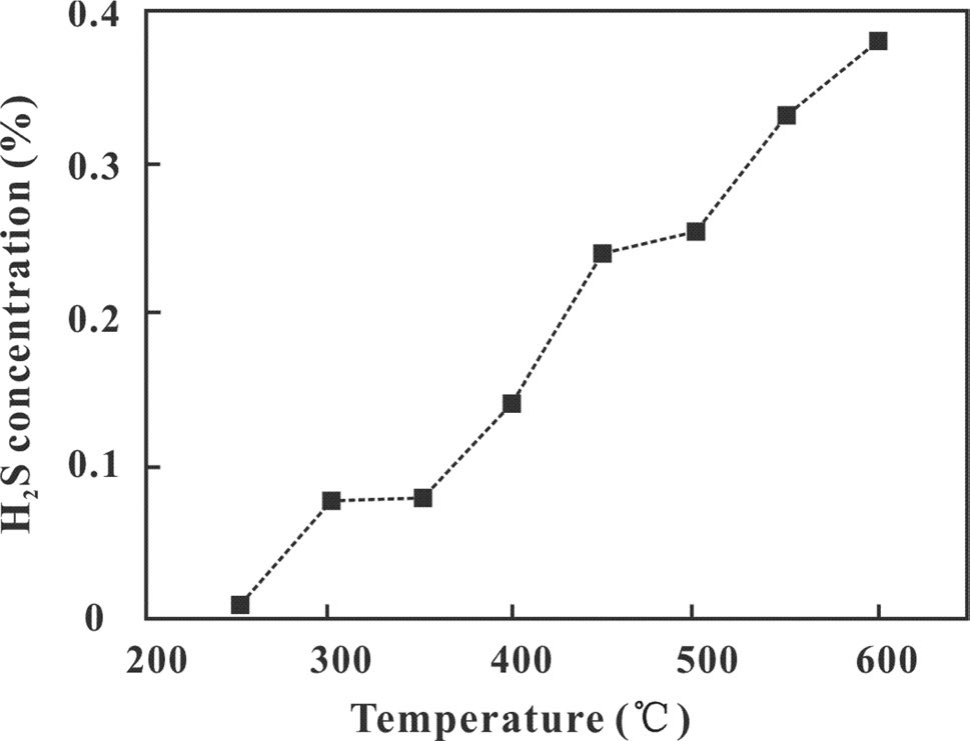
Correlation between temperature and H_2_S concentration.

The degree of reaction was closely related to temperature in the TSR genesis simulation. When the temperature was between 250 °C and 350 °C, the H_2_S concentration in the reactor was less than 0.1% Fig. 3. The concentration increased slowly with an increase in temperature. When the temperature was between 350 °C and 600 °C, the rate of increase in H_2_S concentration with temperature increased, causing the slope of the curve to steepen obviously. It is suggested that when the temperature was relatively low in the 250–350 °C stage, H_2_S mainly formed through the decomposition of organic sulfur in the coal into H_2_S in coal, and the TSR reaction was relatively weak. It is speculated that less H_2_S was produced at this stage, controlled by the total sulfur content in coal seam Table 1. In the 350–600 °C range, the TSR reaction became dominant, and the amount of H_2_S gas in the reactor increased rapidly, reaching a maximum value of 0.38% at 600 °C.

## Discussion

### H_2_S genesis

#### BSR genesis

BSR requires three basic material conditions: organic matter, a sulfur source, and SRB. The main reaction processes proceed according to Eqs. (1) and (2)^6,24–27^.

1 $$ {\text{2C}} + {\text{ SO}}_{4}^{2 - } + {\text{ 2H}}_{{2}} {\text{O}} \to {\text{H}}_{{2}} {\text{S}} + {\text{HCO}}_{3}^{ - } $$

2 $$ {\text{2H}}_{{2}} {\text{S}} + {\text{ Fe}}^{{{2} + }} \to {\text{FeS}}_{{2}} + {\text{2 H}}^{ + } $$

The geochemical results for the coal seam water geochemistry show that the water samples were rich in SO_4_^2−^, the bacterial culture experiments showed abundant SRB in the groundwater from the abnormal areas, and aggregated organic matter is available in the form of coal. Thus, the material conditions in the study area meet the requirements for BSR action. Apart from in coal fire areas, the temperature of the coal seam is generally lower than 50 °C, and the pH value of the water is between 6.8 and 8.4, conditions under which SRB have strong activity. The above analysis suggests that BSR genesis is one of the main causes of certain regions having abnormal concentrations of H_2_S in the study area.

#### TSR genesis

Three basic material conditions are also needed for TSR: organic matter, a sulfur source, and a temperature of coal thermal evolution of more than 90 °C, corresponding to a thermal maturity of organic matter (*R*o) greater than 1.2%, approximately a coal rank above coking coal. The main reaction processes involved are shown in Eqs. (3) and (4)^6,23,28–30^.

3 $$ {\text{2C}} + {\text{ CaSO}}_{{4}} + {\text{ H}}_{{2}} {\text{O}} \to {\text{CaCO}}_{{3}} + {\text{H}}_{{2}} {\text{S}} + {\text{ CO}}_{{2}} $$

4 $$ \sum {\text{CH}} + {\text{ CaSO}}_{{4}} \to {\text{CaCO}}_{{3}} + {\text{H}}_{{2}} {\text{S }} + {\text{ H}}_{{2}} {\text{O}} $$

The *R*_o_ of the coal in the study area ranges from 0.506% to 0.819%, corresponding to a coal rank dominated by gas coal and gas-fertilized coal. The maximum temperature corresponding to this thermal evolution stage is less than 90 °C. Thus, generally, conditions are not appropriate for the formation of H_2_S via TSR. However, due to the exposure conditions of the coal seam in the study area, it undergoes ubiquitous spontaneous combustion, and many such areas are still burning now. Especially in the middle part of the coalfield, the coal seam thickness is large and spontaneous combustion is serious (areas delineated with red dotted lines in Figs. 10–17. Most of the spontaneous combustion occurs at depth of less than 100 m, 226.80 m at a maximum. Spontaneous combustion in a coal seam bakes the surrounding rock at high temperature, causing metamorphism and deformation, and also emits heat and energy to the surroundings. Enough heat (The temperature of coal fire area is higher than 600 °C) is generated by spontaneous combustion of a coal seam to meet the temperature (The temperature needs to higher than 350 °C which obtained from the TSR simulation experiments) conditions of TSR. Meanwhile there are abundant sulfate Table 2 and organic matter, which indicating that TSR genesis in the coal fire areas and adjacent to them is important cause of the development of abnormal H_2_S concentrations.

### Factors influencing H_2_S concentration

#### Relationship between gas composition and H_2_S concentration

**Figure 4:**
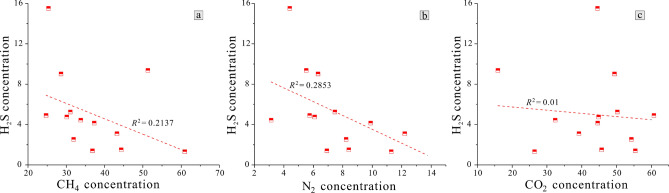
Correlation between gas composition and H_2_S concentration. (**a**) is the correlation of H_2_S concentration versus CH_4_ concentration, (**b**) is the correlation of H_2_S concentration versus N_2_ concentration and (**c**) is the correlation of H_2_S concentration versus CO_2_ concentration.

Lu^31^ concluded that the composition and content of coal mine gas have certain influences on its H_2_S content. In this investigation, H_2_S concentration was found to be negatively correlated with CH_4_ concentration (*R*^2^ = 0.2137), CO_2_ concentration (*R*^2^ = 0.2853), and N_2_ concentration (*R*^2^ = 0.01) Fig. 4a–c. The results of BSR simulation showed that there was a large number of SRB in abnormal-area bottle, but fewer methanogens than in normal-area bottle and that there was a large number of methanogens, but fewer SRB in normal-area bottle. This indicated that SRB and methanogens inhibited each other and hence the presence of large amounts of methane is not conducive to the formation of BSR-genesis H_2_S. BSR produces the reaction products H^+^ and HCO_3_^+^ formation (Eqs. (1) and (3), and CO_2_ dissolved in water will ionize a certain amount of H^+^ and HCO_3_^−^,moreover, CO_2_ is a reaction product in TSR formation. Therefore, the presence of CO_2_ leads to a shift of the reaction equilibrium to the left and proceeds in the direction of the reactants, which is not conducive to the formation of H_2_S. In addition, N_2_ is relatively chemically stable, and is often used as a protective gas, so a high N_2_ content is not conducive to the reaction either.

#### Relationship between coal quality and H_2_S concentration

**Figure 5:**
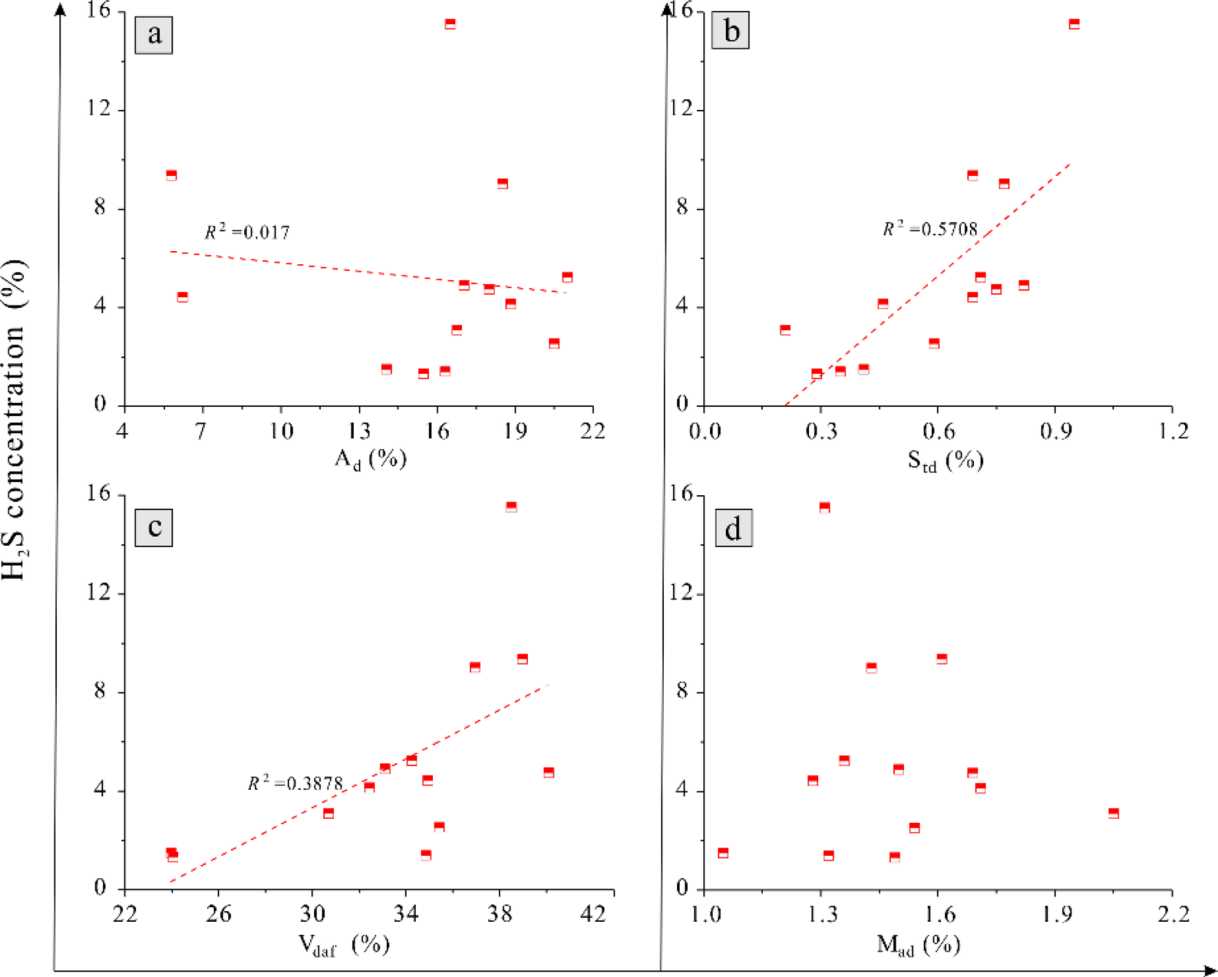
Correlation between coal quality and H_2_S concentration. (**a**) is the correlation of H_2_S concentration versus A_d_ yield, (**b**) is the correlation of H_2_S concentration versus S_td_ content, (**c**) is the correlation of H_2_S concentration versus V_daf_ yield and (**d**) is the correlation of H_2_S concentration versus M_ad_.

Xue^32^ suggested that the content of H_2_S is different in areas with different coal quality characteristics. In this work, the concentration of H_2_S negatively correlated with ash yield (*R*^2^ = 0.017) Fig. 5a, positively correlated with total sulfur content (*R*^2^ = 0.5708) and volatile yield (*R*^2^ = 0.3878) Fig. 5b,c, and had no obvious relationship with water content Fig. 5d. It is considered that the reasons for the above are as follows. The ash in coal is mainly composed of inorganic minerals, mostly carbonate and clay minerals. Carbonate minerals dissolved in water will form a large number of H^+^ and HCO_3_^−^ ions, causing shift of BSR reaction to the left. Also, where massive micron-nanometer scale pores have developed in clay minerals, some H_2_S will exist in the adsorbed state^33^. Volatiles represent the non- aromatic fraction of coal and are mainly composed of gases. Most of them exist in the adsorbed or free state at the surface of the coal matrix or in pores, occupying a large amount of storage space and specific surface area^34^. There is therefore a competitive adsorption relationship between volatiles and H_2_S in the coal matrix and pores. Total sulfur is the direct source of sulfur in H_2_S, and the two values are thus closely related. However, moisture content has no single effect on H_2_S content: on the one hand, water is conductive to the formation of H_2_S as a reactant and reaction medium, but on the other hand, water competes with H_2_S in the coal matrix. The combination of these two effects results in a lack of clear overall correlation between moisture content and H_2_S concentration.

#### Relationship between groundwater geochemistry and H_2_S concentration

**Figure 6:**
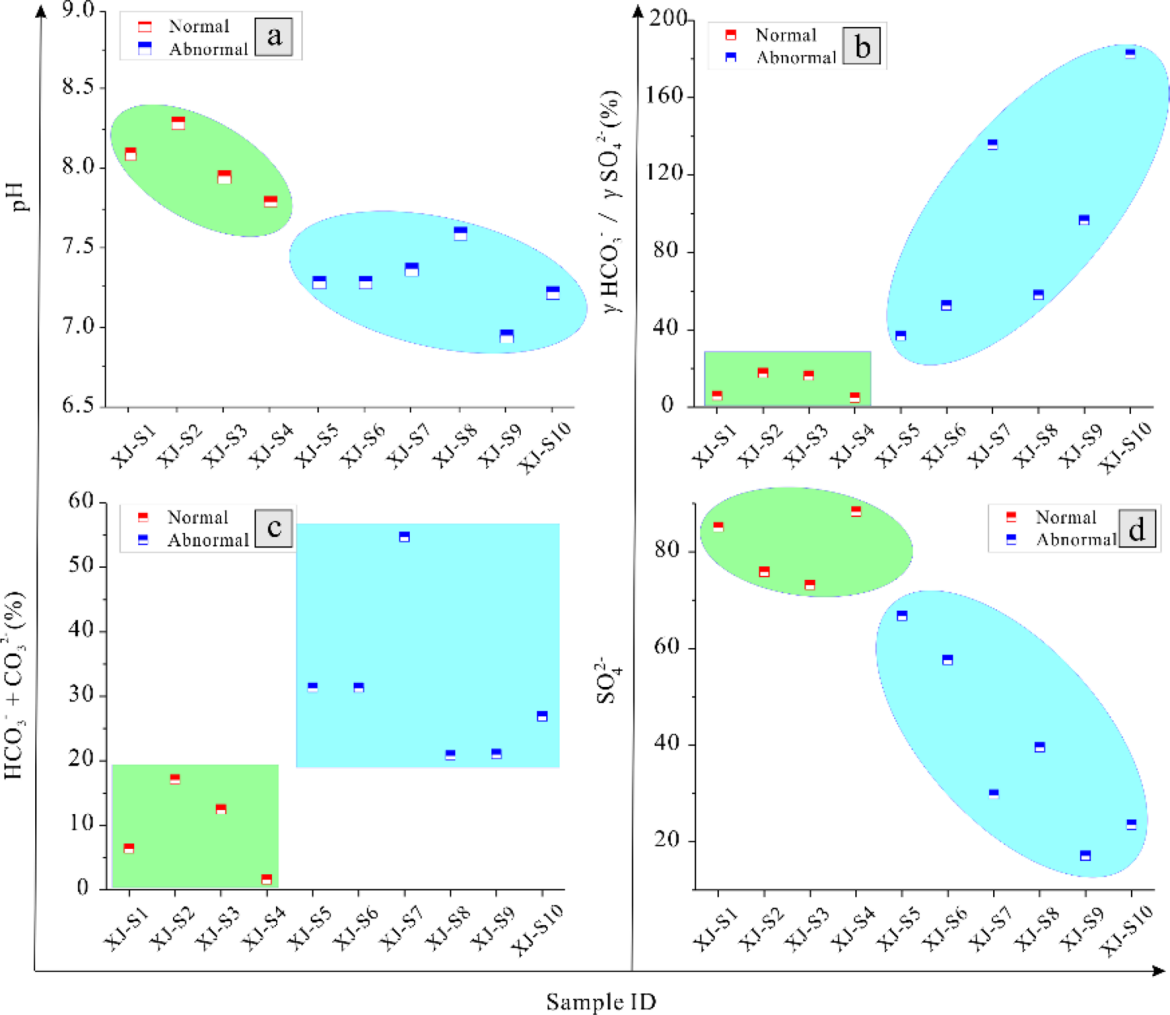
Correlation between groundwater geochemistry and H_2_S concentration. (**a**) The pH of samples, (**b**) is the value of γHCO_3_^−^/γSO_4_^2−^ of samples, (**c**) is the content of HCO_3_^−^ + CO_3_^2−^ and (**d**) is the content of SO_4_^2−^.

Differences in the ion composition, content and pH value of water have important influences on the BSR and TSR reaction processes and control the formation of H_2_S^31,32^. In this study, The SO_4_^2−^ concentration was found to be lower Fig. 6a and the HCO_3_^−^ + CO_3_^2−^ concentration and γHCO_3_^−^/γSO_4_^2−^ to be higher Fig. 6b,c in groundwater from abnormal areas than that from normal areas, while the two had similar pH values Fig. 6d. SO_4_^2−^, as the major source of sulfur will be consumed heavily in the course of the BSR and TSR reactions, causing its depletion in the abnormal areas. CO_2_, a product of the BSR and TSR reactions, exists in the form of HCO_3_^−^ and CO_3_^2−^ after dissolving in water (mainly the former), resulting in a higher HCO_3_^−^ + CO_3_^2−^ concentration in the abnormal areas. As, of SO_4_^2−^ and HCO_3_^−^, one is a reactant and the other a product of the BSR and TSR reactions, the value of γHCO_3_^−^/γSO_4_^2−^ to some content represents the degree of BSR and TSR reaction. So γHCO_3_^−^/γSO_4_^2−^ is generally higher than 50% or even as high as 180% in the abnormal areas, while it is always lower than 20% in the normal areas.

**Figure 7:**
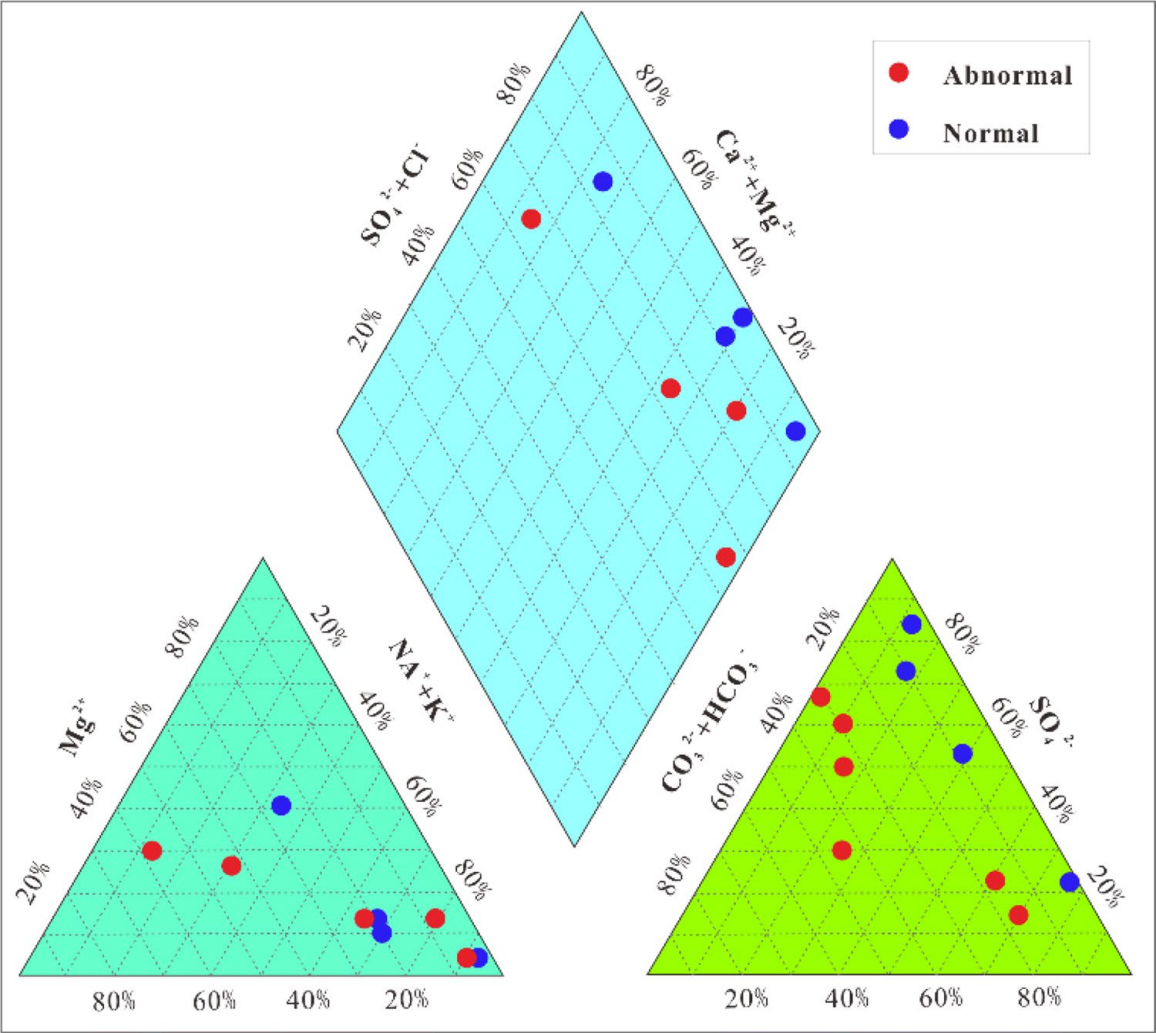
Piper ion diagram for coal seam water samples.

A piper diagram was used to visualized the results for the ion concentration in the coal seam water samples Fig. 7. The Cl^−^ concentration was lower in abnormal area than in normal areas, as was the Cl^−^ + SO_4_^2−^ concentration. Overall, the water type is Na^+^- K + – Cl^−^–SO_4_^2−^—HCO_3_^−^ water in the abnormal areas.

#### Relationship between geological factors and H_2_S concentration

Previous researchers have found that the geological factors controlling H_2_S enrichment are similar to those affecting enrichment in coal bed methane^4,8,10^. The formation and accumulation of H_2_S are mainly controlled by geological factors such as coal seam buried depth, lithological conditions, tectonic conditions, hydrogeological conditions, and the distribution of coal fire areas.

##### Coal seam buried depth

**Figure 8:**
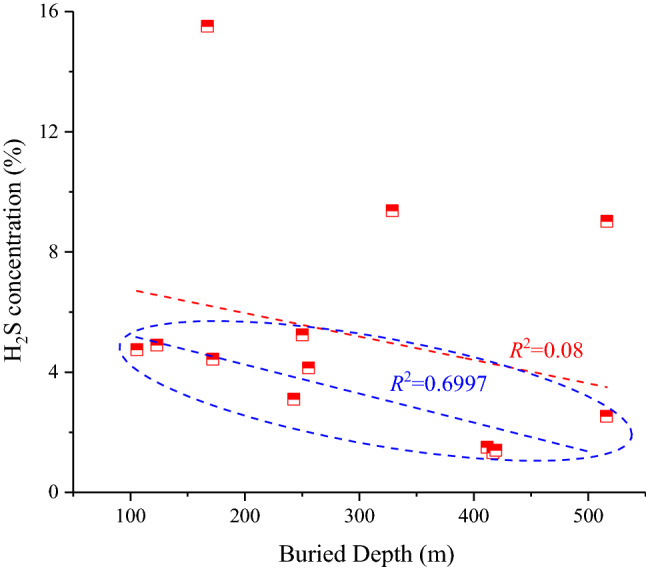
Correlation between buried depth and H_2_S concentration. Where the red fitting line represents all data correlation and the blue fitting line represents the data correlation inside the ellipse.

The H_2_S concentration was weak negatively correlated with coal seam buried depth (*R*^2^ = 0.08), however, the data concentrated in the lower part of the figure (inside the blue ellipse) shows an obvious linear positive correlation (*R*^2^ = 0.6997). On the whole, therefore, the buried depth has an inhibitory effect on the concentration of H_2_S in coal mine gas. In that H_2_S concentration decreased with an increase in buried depth Fig. 8. Overburden formation pressure increases as buried depth increases, and the adsorption capacity of a rock matrix is controlled by pressure: the higher the pressure, the stronger the adsorption capacity^34^. Thus, in seams at depth, H_2_S mostly occurred on the surface of the coal matrix in the absorbed state, which resulted in a decrease in H_2_S in coal mine gas.

##### Lithological conditions

The lithological character of the cap rock is one of the main parameters for gas reservoir evaluation^35,36^. The main mineable coal seams in the study area are Jurassic continental coal-bearing strata of the Badaowan Formation (J_1_b) and the Xishanyao Formation (J_2_x). The bility of overlying Cretaceous and Cenozoic cap rocks provide a seal to gas reservoirs is of great importance. The cap rock in the study area performs well and has a wide extent and consistent thickness. It is mostly Paleogene gypsum and gypsum-mudstone, Cretaceous mudstone and Jurassic mudstone. Which is compact and generally has low porosity and permeability, providing stable lithological traps with a good sealing effect.

##### Tectonic and hydrogeological conditions

Xishan Mine takes the forms of an east–west extending narrow, irregular strip Fig. 1. It is located in the front fold zone of the Bogda thrust nappe between the Fukang and Yaomoshan faults, which is arc-shaped with a northward bulge. Mesozoic faults and folds dominate the area, most are compressive-torsional making them conductive to good sealing^10,16^, and the H_2_S enriched areas are most concentrated at the tectonic high-point of the arc part of the nappe, conforming to the characteristics of syncline controlled gas and reservoir formation at tectonic high points. In addition, the hydraulic connection between coal aquifers and other aquifers is weak, and the direction of gas migration is opposite to that of groundwater flow^10,16^, which has played a role in promoting hydraulic plugging.

##### Coal fire areas

H_2_S enriched areas are generally located in coal fire areas and their peripheries. Coal fire mainly affects the formation and enrichment of H_2_S in two ways. One is that the spontaneous combustion of coal seams produces a large amount of energy, which radiates to the surrounding area, resulting in an increase in overall energy and temperature in the area, and creating conditions that are favorable to the formation of TSR-genesis H_2_S. Moreover, metamorphism occurs in rocks baked at high temperature for a long time near coal fire areas, and partial melting and recrystallization of minerals will fill the original cracks in the rock, blocking the escape of H_2_S.

### Prediction of spatial variation in H_2_S concentration

Analysis of the results of gas chromatography of coal mine gas can quantitatively characterize the H_2_S concentration in different strata and working faces of the Xishan mine area. The above research shows that H_2_S concentration has complex relationship with the groundwater geochemistry, the physical properties of the coal and various geological factors. These relationships can be summarized as follows. The H_2_S concentration is linearly positively correlated with volatile yield and total sulfur content, negatively correlated with CH_4_ concentration, N_2_ concentration, CO_2_ concentration and ash yield. Moreover, compared with non-anomalous areas, anomalous areas have lower SO_4_^2−^, higher HCO_3_^−^ + CO_3_^2−^ content, and a higher γ HCO_3_^−^/γ SO_4_^2−^ ratio. To predict H_2_S concentration and intuitively characterize the distribution of areas with abnormally high H_2_S, the analytic hierarchy process (AHP) and MAPGIS (MAPGIS 10.3, manufactured by China University of Geosciences and Engineering Research Center of GIS software and its application Ministry of Education (Zondy Cyber Co., Ltd, Wuhan, China), URL link: http://www.mapgis.com/) were used to establish an image model of H_2_S anomaly concentration that takes into account the process of generation, migration and preservation of H_2_S together with the effects of gas composition, coal quality characteristics, groundwater geochemistry, and other geological factors.

#### Establishment of analytical hierarchy process model

**Figure 9:**
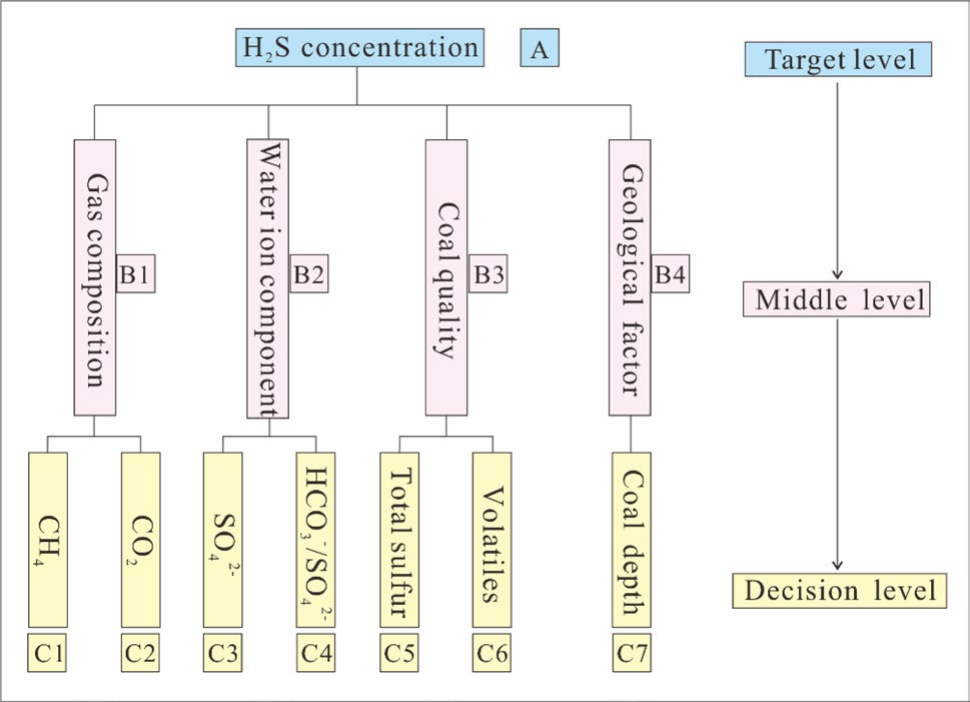
Schematic diagram of the AHP structure model.

AHP model can take various decision-making factors into account qualitatively and quantitatively and has been widely used for in dealing with multi-factor comprehensive evaluation^37,38^. It is a step-by-step transfer model based on the specific conditions of the research object and generally includes three levels: the target level, the middle level, and the decision level. The research model is shown in Fig. 9. According to experimental analysis and single factor fitting results, H_2_S concentration is taken as the target level of the model, and the gas composition, groundwater geochemistry, coal quality and other geological factors are taken as the middle level, CH_4_ concentration, CO_2_ concentration, SO_4_^2−^ concentration, γ HCO_3_^−^/γ SO_4_^2−^, total sulfur content, volatile yield and sampling depth are taken as the decision level, each of which corresponds with one of the middle levels. The model was thus built by systematically analyzing the research objectives are analyzed systematically, establishing several influencing factors, and then determining the secondary influencing those primary factors. In this way, the factors that affect each other and act upon each other were decomposed step by step, forming a top-down hierarchical relationship structure. When determining the factors, it is important to follow the principle of highlighting the main indicators, the principle of the relative independence of the main factors and the principle of operability in the establishment of each factor.

#### Establishment of hierarchical matrix

The hierarchical structure reflects the relationships among various factors, but each factor differs in its degree of influence on the target measurement. After referring to previous research^1,6,10,23,24,36,37^, this study assigns each factor according to Saaty's 9-level assignment theory and establishes a hierarchical matrix Tables 3, 4, 5 and 6. The weight calculation criteria of the AHP model are then used to obtain the maximum eigenvalue λ_MAX_ and its corresponding eigenvectors according to the obtained matrix. The normalized eigenvectors are the single ranking weight W of each matrix. The total ranking weight WA/Ci is then calculated according to the transferability of the matrix. The results are given in Table 7.

**Table 3:** Judgment matrix A-Bi (i = 1–4).

A	B1	B2	B3	B4
B1	1.00	2.00	0.50	1.00
B2	0.50	1.00	0.25	1.00
B3	2.00	4.00	1.00	2.00
B4	1.00	2.00	0.50	1.00

**Table 4:** Judgment matrix B1-Ci (i = 1–2).

B1	C1	C2
C1	1.00	0.50
C2	2.00	1.00

**Table 5:** Judgment matrix B2-Ci (i = 3–4).

B2	C3	C4
C3	1.00	0.50
C4	2.00	1.00

**Table 6:** Judgment matrix B3-Ci (i = 5 ~ 6).

B3	C5	C6
C5	1.00	0.25
C6	4.00	1.00

**Table 7:** Weight of main factors controlling H_2_S concentration.

Controlling factors	CH_4_ concentration (W_1_)	CO_2_ concentration (W_2_)	SO_4_^2−^ concentration (W_3_)	γSO_4_^2−^/γHCO_3_^−^(W_4_)	Total sulfur concentration (W_5_)	Volatile yield (W_6_)	Sampling depth (W_7_)
Weight (Wi)	0.024	0.145	0.045	0.089	0.087	0.346	0.217

#### Single-factor thematic map and multi-factor superposition map predictions

The superposition analysis function of a MAPGIS database is used to compound all of the information is compounded into a comprehensive information that relevant to the partition model of H_2_S concentration in the coal mine. That is to say, a mathematical model is established that correlates the H_2_S concentration and its influencing factors. The values calculated by the model directly reflect the risk that there will be an abnormal H_2_S concentration at a certain geographical location. The model is based on gas composition, groundwater geochemistry, coal quality, and other geological factors, and, using the results of the hierarchical analysis, weights are assigned to each factor to make a comprehensive multi-factor prediction. The RI (Risk Index) is introduced to evaluate the risk, which is defined as the sum of the superimposed effects of various factors on the H_2_S concentration in a certain area (Eq. (5)).

5 $$ {\text{RI}} = \mathop \sum \limits_{k = 1}^{n} W_{k} *f_{k} \left( {x,y} \right) $$

where RI is the risk index; W_k_ is the weight of the dominant factor; f_k_ (x, y) is the function of a single-factor influence value; x, y is geographical coordinate; n is the number of influencing factors.

**Figure 10:**
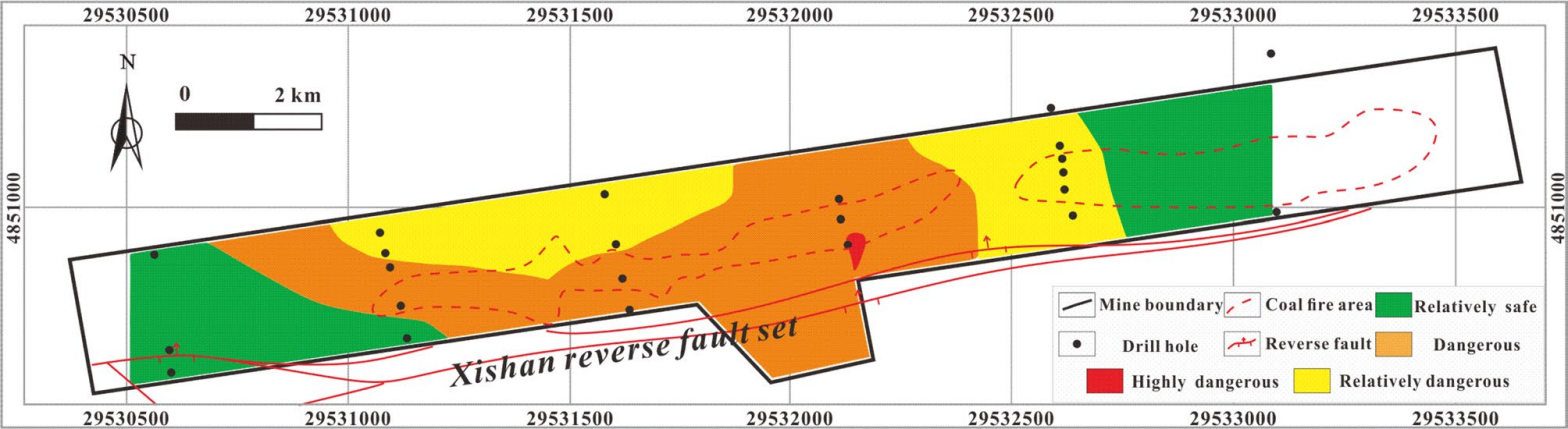
Thematic map of CH_4_ concentration in MAPGIS software.

**Figure 11:**
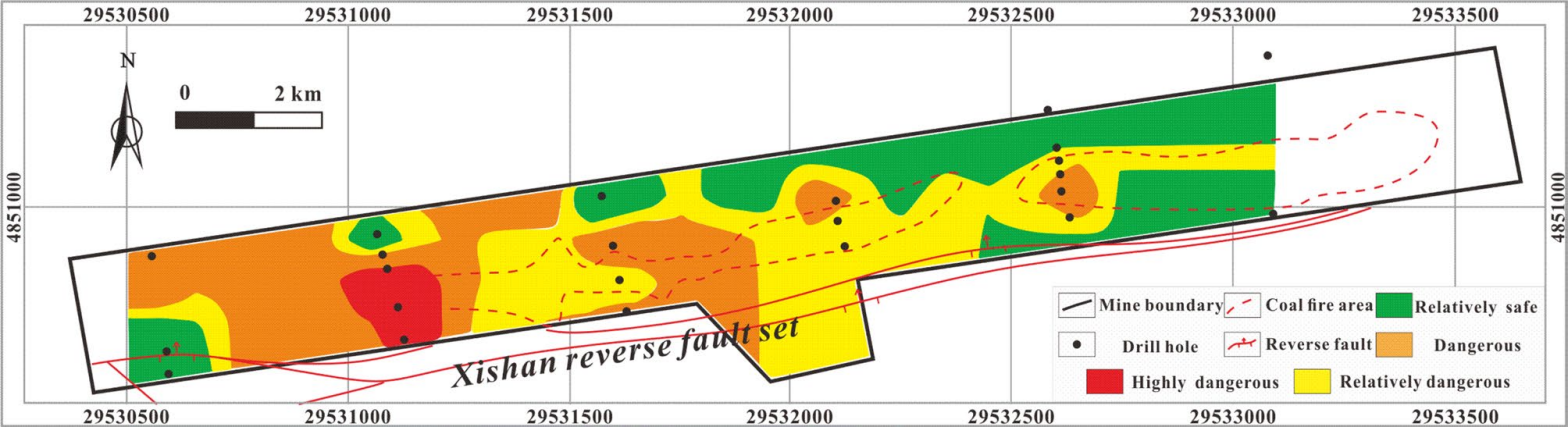
Thematic map of CO_2_ concentration in MAPGIS software.

**Figure 12:**
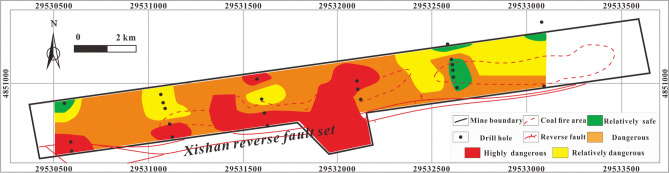
Thematic map of SO_4_^2−^ concentration in MAPGIS software.

**Figure 13:**
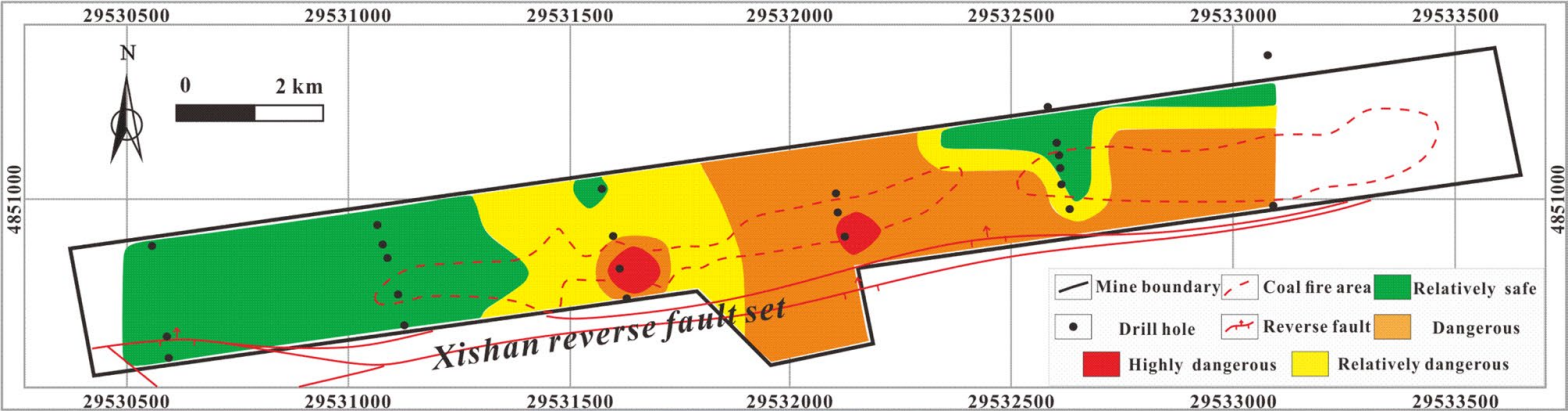
Thematic map of γHCO_3_^−^/γSO_4_^2−^ in MAPGIS software.

**Figure 14:**
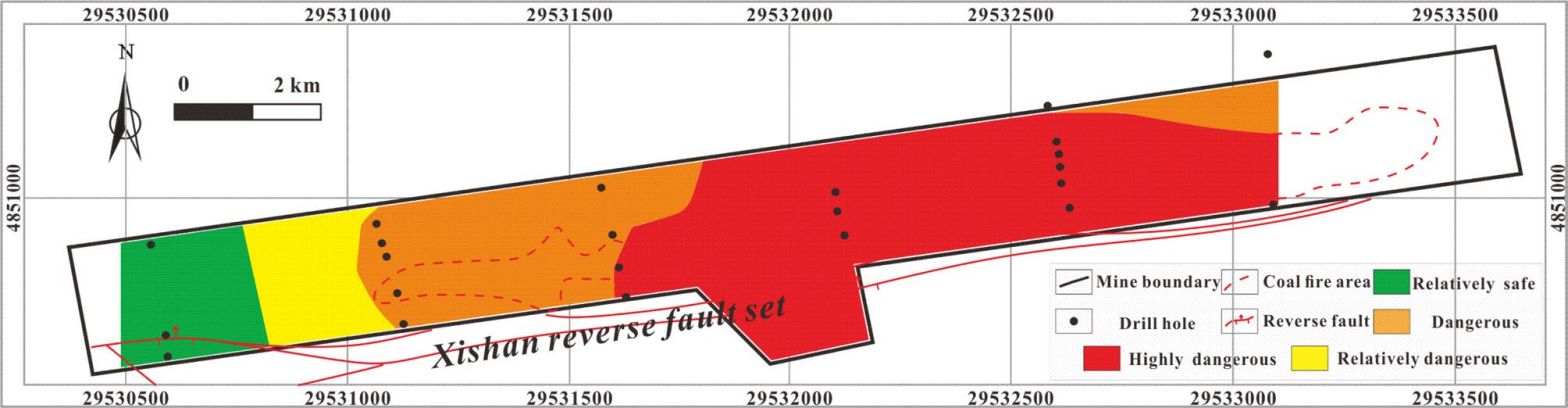
Thematic map of total sulfur concentration in MAPGIS software.

**Figure 15:**
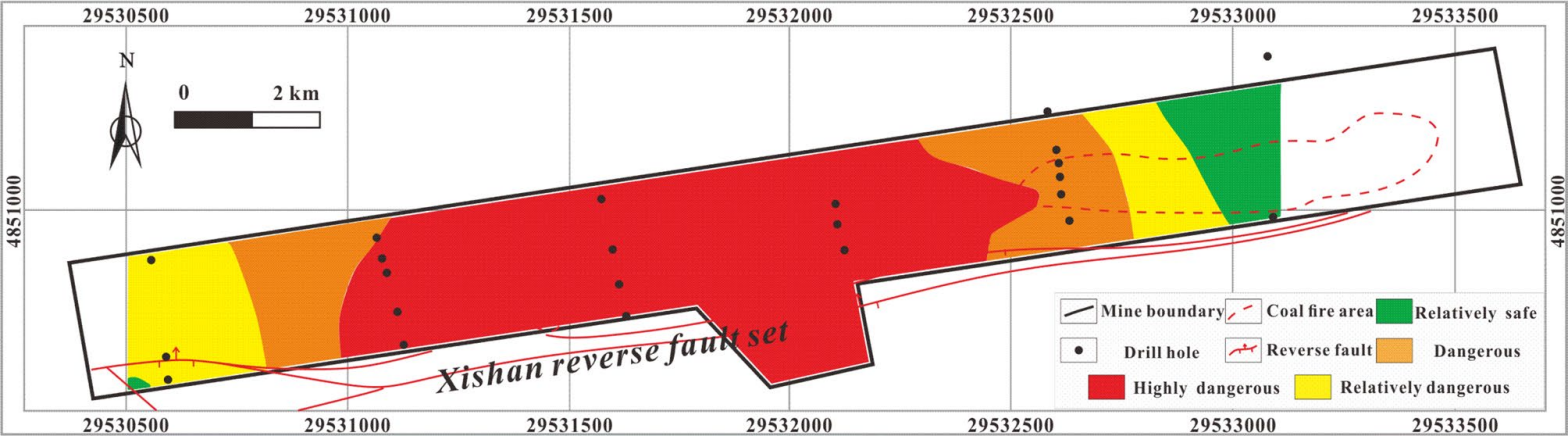
Thematic map of volatile yield in MAPGIS software.

**Figure 16:**
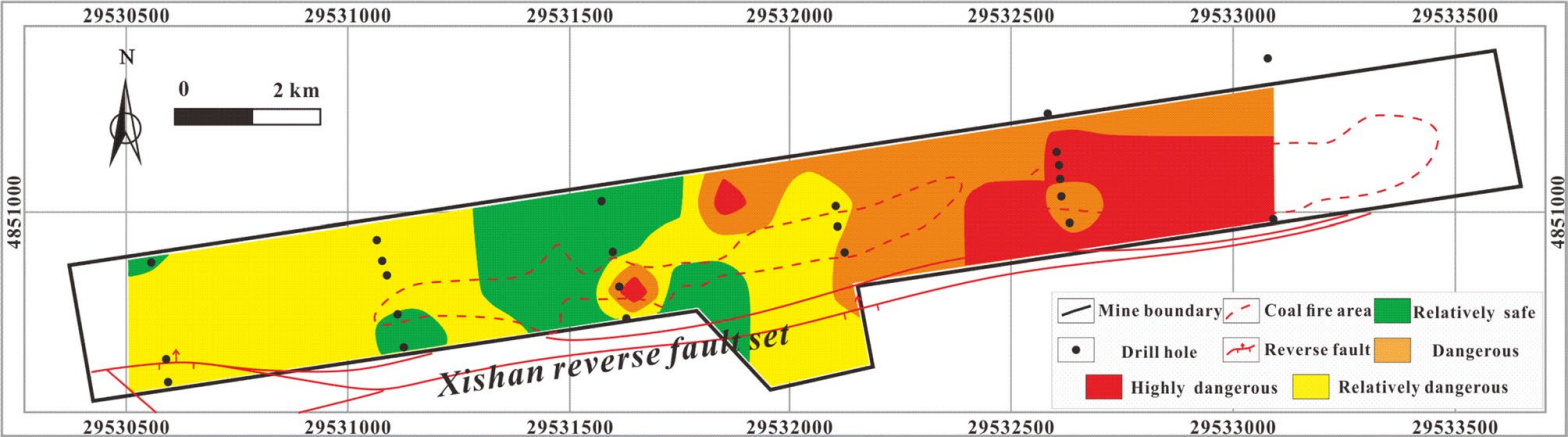
Thematic map of sampling depth in MAPGIS software.

**Figure 17:**
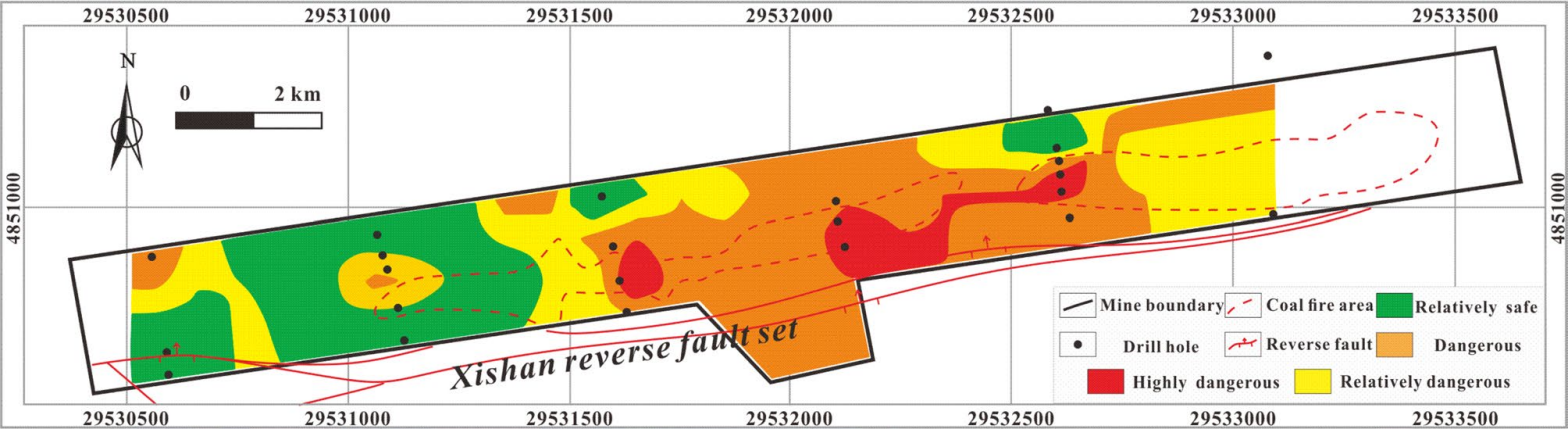
Thematic map of multi-factor superposition in MAPGIS software.

The coordinates of the drill hole and the quantified values should be input into the MAPGIS to predict the RI coupling MAPGIS and AHP. The powerful data storage, spatial data processing and analysis functions, results display and output functions and data update functions of MAPGIS are then used to generate corresponding data files that can be read by drawing software. These are then processed by mesh generation and interpolation. Finally, the quantified results are displayed in the form of graphics, and the result is output as a diagram through the graphics output system. According to the normalized value of the H_2_S concentration gradient, the natural grading method (also known as the best difference method) can be used to divide the study area into highly dangerous, dangerous, relatively dangerous and relatively safe areas, which correspond to different colors. The darker the color, the higher the risk coefficient. RI values within the range 0–0.000015% are safe area (green), an RI ranging 0.000015–0.00036% is relatively dangerous (yellow), an RI ranging from 0.00036 to 0.0023% is dangerous (orange), and an RI ranging 0.0023–0.015% is highly dangerous (red) Figs. 10–17.

The thematic map of the single influencing factor of CH_4_ concentration Fig. 10 shows that the highly dangerous area is located in the central coal fire area of the mining area and is very small. Most of the study area is relatively safe or relatively dangerous apart from in and around the central coal fire area.

The thematic map of the single influencing factor of CO_2_ concentration Fig. 11 shows that a highly dangerous area in the southwest corner of the central coal fire area and dangerous areas distributed around the highly dangerous area and in the coal fire areas. The relatively safe and relatively dangerous areas are mainly in the eastern part and in a small area in the southwest of the mining area.

The thematic map of the single influencing factor of SO_4_^2−^ concentration Fig. 12 shows that the main body of highly dangerous area is located in the central coal fire area and that certain regions of the west are also highly dangerous, while the dangerous area occupies most of the study area. The relatively safe and relatively dangerous areas are mainly located in the northeast of the study area as well as an isolated part of the central and western areas.

The thematic map of the single influencing factor of γHCO_3_^−^/γSO_4_^2−^ Fig. 13 shows that the highly dangerous area is relatively small and is concentrated in the central coal fire area. The main part of the dangerous area is located in the coal fire area and its surroundings, while the relatively safe and relatively dangerous areas are mainly located outside the coal fire areas.

The thematic map of the single influencing factor of sulfur concentration Fig. 14 shows that the coal fire areas and the areas surrounding them are all high-danger or dangerous, while the western area is relatively safe or relatively dangerous.

The thematic map of the single influencing factor of volatile yield Fig. 15 shows a stepped downward trend in the H2S concentration from the middle to either side of the study area. The highly dangerous and dangerous areas are located in the middle part, while the relatively safe and relatively dangerous areas are located at the east and west margins.

The thematic map of the single influencing factor of sampling depth shows Fig. 16 that the highly dangerous area is mainly in the east coal fire area and in certain parts of the central coal fire area. The dangerous area is mainly located in the northeastern part of the study area, while relatively safe and relatively dangerous areas occupy the central and western parts of the study area.

The thematic map of a single influencing factor can reflect the how that variable controls the distribution of H_2_S under specific conditions, but a thematic map with multi-factor superposition can predict the characteristics and variability of overall H_2_S concentration under the control of multiple factors accurately. The multi-factor thematic map is shown in Fig. 17. The highly dangerous area is small: it is mainly located in the eastern coal fire area, with a few highly dangerous areas in the central coal fire area. The dangerous area is mainly located on both sides of the area between the two coal fire areas. The relatively safe and relatively dangerous areas are mainly in the west and northeast. The prediction results are relative accurate: several H_2_S disasters have occurred in the history of the study area and all were in areas indicated in Fig. 17 to be highly dangerous or dangerous.

## Conclusion

1. Disasters resulting from abnormal concentration of H_2_S have occurred in many coal seams and working faces at the Xishan coal mine. The H_2_S is derived from two processes, BSR and TSR. Two genera of SRB are primarily responsible for the BSR, *Desulfovibrio* and *Desulfomicrobium*, and the TSR-genesis of H_2_S, which requires temperature, mainly occurred in coal fire areas.
2. The H_2_S concentration is negatively correlated with CH_4_ concentration, N_2_ concentration, CO_2_ concentration and ash yield and positively correlated with volatile yield and total sulfur content. Moreover, the SO_4_^2−^ concentration is lower and HCO_3_^−^ + CO_3_^2−^ and γHCO_3_^−^/γSO_4_^2−^ are higher in areas with an abnormal H_2_S concentration.
3. The overall tectonic conditions of the study area are simple, with the cap rock lithology and tectonic and hydrogeological conditions being conductive to the storage of H_2_S. In addition, the presence of two coal fire areas is favorable to the formation of H_2_S.
4. An Analytical Hierarchy Process Model and the results of MAPGIS prediction show that areas that can be considered highly dangerous and dangerous as regards H_2_S concentration are mainly located in and around the coal fire areas, while the west and northeast of the study area can be considered relatively safe to relatively dangerous.
